# Quality and reliability of YouTube videos on fascial sling surgery

**DOI:** 10.1590/1806-9282.20250140

**Published:** 2025-08-08

**Authors:** Ahmet Emin Doğan, Emre Hepsen, Hilmi Sari

**Affiliations:** 1 Etlik City Hospital – Ankara, Turkey.

## INTRODUCTION

Female urinary incontinence (UI) is a common health problem that significantly impacts the quality of life for many women. The most prevalent form is stress UI (SUI), characterized by involuntary urine loss triggered by activities that increase intra-abdominal pressure, such as sneezing, coughing, or exercising^1,2^. When conservative treatments are ineffective, surgical interventions become necessary. Among these, fascial sling procedures have gained popularity due to their established long-term success rates and lower complication rates compared to synthetic options^3,4^. The fascial sling technique utilizes autologous tissue—usually harvested from the rectus fascia or fascia lata—to create a supportive sling under the urethra, which improves urinary control by providing structural support^5^. This method is often recommended for patients experiencing recurrent SUI or for whom synthetic materials pose a higher risk. Although the clinical outcomes are generally positive, fascial sling surgery is technically demanding and requires comprehensive preoperative counseling, patient education, and collaborative decision-making among healthcare providers and patients.

Given the complexities and potential complications linked to fascial sling procedures, it is essential for patients to access accurate and trustworthy information. With the growing trend of online health research, patients frequently seek information from platforms like YouTube. YouTube has emerged as a widely accessible platform for health-related information due to its user-friendly interface and the ability to provide visual and auditory content. However, the accuracy and reliability of information found on such platforms can vary significantly, as content is not subject to systematic review or regulatory oversight^6^. While prior studies have assessed the quality of YouTube videos on other urological procedures, there has been no focused evaluation of the quality and reliability of videos pertaining specifically to fascial sling surgery for female UI^7,8^. The study used YouTube because it has gained popularity as a source of health information, particularly for visual and procedural content.

This study primarily aims to evaluate the quality and reliability of YouTube videos about fascial sling surgery for female UI to assist both patients and clinicians in identifying credible information sources.

## METHODS

As the study utilized only publicly available data and did not involve human subjects, approval by an ethical committee was not necessary. A systematic search of YouTube was conducted on June 1, 2024, using the search term "fascial sling surgery" to find videos related to the surgical management of female UI. The search was executed in an incognito browser to prevent any bias related to browsing history or cookies. Only English-language videos uploaded within the last 5 years were considered for the analysis.

The initial search results were filtered based on "relevance," and duplicate entries were merged into a single video. The initial results obtained upon inputting the terms were "filtered" by "relevance," i.e., rankings by the algorithm of YouTube based on views, likes, recency, and user engagement. This process ensured that those that were most viewed and engaged would be considered for the study. A total of 50 videos were chosen for evaluation, as this sample size is commonly used in similar urology studies^9,10^. Videos were excluded if they were not in English, unrelated to fascial sling surgery, duplicates, contained inappropriate content, or lacked audio.

Notably, two independent reviewers, both experienced urologists, evaluated each selected video. Both reviewers possessed extensive experience with fascial sling procedures and reviewed the European Association of Urology (EAU) Guidelines on Non-Neurogenic Female Lower Urinary Tract Symptoms (2024 edition) to ensure a thorough understanding of the topic prior to the evaluation^11^. For each video, the duration (in seconds) and the number of days since it was uploaded were recorded. Video creators were categorized as academic healthcare professionals, non-academic healthcare professionals, physicians, nurses, patients, or others. Additionally, data on views, likes, and comments were documented.

Video quality was assessed using a five-point scoring system established by Bernard et al. to evaluate online health information. The GQS classifies content quality from 1 (very poor quality) to 5 (excellent quality), with scores of 4 or 5 indicating high-quality resources, a score of 3 reflecting moderate quality, and scores of 1 or 2 signifying low-quality content^12^.

To assess video reliability, the modified DISCERN tool was applied. This tool comprises five binary (yes/no) questions, with each "yes" earning 1 point, yielding a maximum score of 5. The modified DISCERN criteria evaluates aspects such as potential bias, objectivity, overall reliability, transparency, and the presence of references or additional resources. Videos receiving a score of 5 were deemed highly reliable, those scoring 3 or 4 as moderately reliable, and those scoring 1 or 2 as having low reliability^13^. A summary of the GQS and modified DISCERN rating systems is provided in Table 1.

**Table 1:** Global quality scale assessment criteria.

GQS score	Description
1	Very poor quality
2	Poor quality
3	Moderate quality
4	Good quality
5	Excellent quality

GQS: global quality scale.

## RESULTS

A total of 50 videos related to fascial sling surgery for female UI were initially identified and analyzed. Five videos were excluded due to lacking audio or being duplicates, which were subsequently replaced by the next five videos in the search results. The average number of views per video was 9,572±21,843, leading to a total of 478,600 views across all videos. The high standard deviation in views simply indicates heterogeneity in the popularity of the videos, where some are viewed orders of magnitude more than others. The average video duration was 418±425 s, with an average of 58 likes per video (range: 0–298).

It is noteworthy that all videos were created by healthcare professionals specializing in female pelvic health, with 42% (21/50) holding academic titles. Additionally, promotional content related to the creator's institution (e.g., private hospitals or clinics) was identified in 8% (4/50) of the videos. The most commonly discussed parameters relevant to patients included surgery duration (68%), anatomical success rates (66%), comparisons of advantages and disadvantages between fascial slings and synthetic slings (64%), and postoperative complications (58%).

### Quality and reliability evaluation

The intraclass correlation coefficient for DISCERN scores was 0.894, while for GQS scores, it was 0.915, indicating a high level of agreement between reviewers. The mean GQS score of the videos was 3.16±0.72. Approximately 6% (3/50) of the videos were identified as having inappropriate or misleading content for patients, particularly in technical details such as the type and placement of the sling, the type and number of sutures used, and the necessity of additional procedures (e.g., cystoscopy). A summary of the analysis of the 50 videos is provided in Table 2.

**Table 2:** Summary of YouTube video analysis.

Assessment criteria	Results
Total number of videos	50
Average number of views	9,572±21,843
Average video duration (seconds)	418±425
Average number of likes	58 (range: 0–298)
Creator's category	100% healthcare professionals
Percentage of creators with academic titles	42%
Percentage of misleading content	6%
Most commonly discussed topics	- Surgery duration (68%)
	- Anatomical success rates (66%)
	- Comparison of fascial sling and synthetic slings (64%)
	- Postoperative complications (58%)

The distribution of GQS scores revealed that 10% (5/50) of the videos were of poor quality and provided limited information, 30% (15/50) were inadequate but contained some relevant content for patients, 40% (20/50) were of adequate quality and provided a satisfactory amount of information for patient education, while 14% (7/50) were rated as excellent, offering comprehensive and accurate details.

The average DISCERN score was 2.63±1.2, indicating a moderate level of reliability overall. None of the videos cited appropriate references, and only 20% (10/50) presented unbiased, balanced discussions devoid of promotional content. Consequently, none of the videos achieved a perfect DISCERN score. Evaluation details are presented in Table 3.

**Table 3:** Quality and reliability assessment of videos.

Evaluation scale	Results
Average GQS score	3.16±0.72
Average modified DISCERN score	2.63±1.2
Percentage of high quality (GQS 4–5)	22% (11/50)
Percentage of moderate quality (GQS 3)	36% (18/50)
Percentage of low quality (GQS 1–2)	10% (5/50)
Percentage of high reliability (DISCERN 5)	0%
Percentage of moderate reliability (DISCERN 3–4)	20% (10/50)
Percentage of low reliability (DISCERN 1–2)	80% (40/50)

GQS: global quality scale.

## DISCUSSION

Many patients turn to online platforms to gather information about their health conditions or treatment options. At this time, technological applications are becoming prominent due to the rising proportion of elderly individuals and chronic diseases, helping public health and healthcare systems remain viable. An experiment has established that the use of virtual reality systems in rehabilitation enhances the quality of life, exercise compliance, and motivation of patients^14,15^.

YouTube is frequently accessed for this purpose and features a diverse array of health-related content. However, similar to non-health-related videos, health-related videos on YouTube may also contain misleading information due to the lack of expert reviews and regulatory oversight^16^. While numerous studies have evaluated the reliability and quality of surgical and internal medical content online^12,17,18^, investigations specifically focusing on fascial sling surgery videos are limited. Our research indicates that although YouTube videos discussing fascial sling surgery have acceptable quality, their reliability is insufficient.

In our study, we selected and analyzed the top 50 most relevant videos. While there is no universally accepted standard regarding the number of videos to include in such studies, previous research has typically used sample sizes of 50 or 100 videos^9,10,18^. The videos chosen for our analysis were among the most viewed and liked, suggesting a strong engagement from urogynecologists on social media.

The average GQS score of the videos was 3.16±0.72, indicating that the overall content quality is acceptable. Specifically, 36% of the videos received a GQS score of 3, reflecting moderate quality with some information gaps, while 34% scored 4, denoting good quality with most essential topics sufficiently covered. Only 8% of the videos were rated as excellent, providing comprehensive content beneficial to patients. In contrast, the average DISCERN score was found to be 2.63±1.2, with none of the videos citing appropriate sources and only 20% presenting balanced, impartial information. Therefore, none of the analyzed videos achieved a full score based on the DISCERN criteria, and 60% were classified as unreliable.

These findings align with existing literature, demonstrating variability in the reliability of health-related YouTube videos. For instance, Gul et al. reported that 30% of videos on premature ejaculation were deemed unreliable^19^, while Tonyali et al. highlighted the inadequate informational value of RIRS videos for patients, attributed to the low percentage of patient-targeted videos (12.7%)^20^. Additionally, Langford et al. assessed that 15% of spinal cord stimulation videos were misleading^21^. Research focusing on urogynecology has yielded comparable results; for example, Batur et al. reported low overall quality and reliability of YouTube videos on overactive bladder, with average GQS and DISCERN scores of 3.4 and 2.6, respectively, in 2022^18^. Our results show that the quality of videos on fascial sling surgery is on par with those addressing other urological and gynecological topics, revealing a widespread issue across diverse health information available on social media.

Our study had some limitations. The first limitation of this study was that it consisted of only the first 50 videos from YouTube's website with the keyword "fascial sling surgery." Our sample size was not very large. However, according to the literature, individuals often search the first few pages for results^22,23^. Therefore, we thought that 50 videos were a sufficient number for interpretation. Second, we limited the study to only English videos. Hence, it was not possible to establish the value of other language videos, which could potentially hold critical information. While the video's interpretation is subjective, we minimized personal bias by having two independent urologists analyze the videos. One more problem is that there isn't a standard way to measure the quality and reliability of video-based medical content. Despite their widespread use, GQS and DISCERN lack specific validation as testing tools. This study is based on the content analysis of searched YouTube videos. However, unsearched content on YouTube, where the overall quality and reliability of information may be far inferior, occasionally exposes patients and others. Therefore, our results may not apply to all health information videos on YouTube. Finally, YouTube's dynamic nature and constant uploading of new content may lead to differences in results over time. Despite these limitations, YouTube continues to be an important source of health information for patients. The videos often serve as an initial point of contact, enhancing awareness and understanding of health issues. Healthcare providers must engage with patients by recognizing their reliance on digital media for health information and guiding them toward credible sources. Improving the quality of educational materials available on platforms like YouTube could empower patients and ensure they receive accurate information regarding their health conditions. To improve the reliability and quality of YouTube videos, health professionals must work with medical institutions to create content that is accurate and engaging. Including references, citations, and links to peer-reviewed studies in video comments would further increase credibility. There are several important aspects of this study that enhance its clinical and academic value. To our knowledge, this study represents the first systematic review of YouTube content specifically focused on fascial sling surgery, which is a technically complex procedure, and patient education is critical for informed decision-making. Our study was consistent with high-level standards, including double independent review by senior urologists, assessment with commonly used measurement tools (GQS and modified DISCERN), and emphasis on the most clinically relevant videos (first 50 videos).

In conclusion, our study underscores the importance of scrutinizing online health information sources, particularly those addressing surgical procedures. While the average quality of YouTube videos discussing fascial sling surgery is moderate, their reliability falls short of satisfactory standards. As healthcare providers, we have a responsibility to support our patients in their pursuit of accurate information and to actively contribute to the creation of high-quality educational resources on platforms they frequently use.

## ETHICS APPROVAL AND CONSENT TO PARTICIPATE

Since no patient data or materials were used and all videos are publicly available on the social media website (youtube.com), there was no need to obtain an institutional review board or ethics committee approval for this study.

## Data Availability

The datasets generated and/or analyzed during the current study are available from the corresponding author upon reasonable request.
