# ERRATUM: Quality and reliability of YouTube videos on fascial sling surgery

**DOI:** 10.1590/1806-9282.20250140-ER

**Published:** 2025-11-03

In the manuscript: Doğan AE, Hepsen E, Sari H. Quality and reliability of YouTube videos on fascial sling surgery. Rev Assoc Med Bras. 2025;71(7):e20250140. https://doi.org/10.1590/1806-9282.20250140

**Where it read:**

Review Article

**It should read:**

Original Article

**On page 1 it was included:**

**SUMMARY**

**OBJECTIVE:** Female urinary incontinence affects many women's quality of life. Access to accurate information is crucial due to the complexities and potential complications of fascial sling procedures. The aim of this study was to evaluate the quality and reliability of YouTube videos related to fascial sling surgery, to guide patients and healthcare providers toward credible sources of information.

**METHODS:** A systematic search was conducted on June 1, 2024, using the term "fascial sling surgery" on YouTube. A total of 50 English-language videos uploaded in the last 5 years were selected based on relevance. Two independent urologists assessed each video using the Global Quality Scale and the modified DISCERN tool to determine content quality and reliability, respectively. The Global Quality Scale scores range from 1 (very poor) to 5 (excellent), while the modified DISCERN tool provides a maximum score of 5 based on five criteria.

**RESULTS:** The analysis revealed a mean Global Quality Scale score of 3.16±0.72, indicating acceptable quality. The mean DISCERN score was 2.63±1.2, reflecting moderate reliability. Approximately 6% of the videos contained misleading information. Most videos were created by experienced healthcare professionals, yet only 8% received an excellent quality rating.

**CONCLUSION:** YouTube serves as a resource for patients seeking information on fascial sling surgery for female urinary incontinence. However, the reliability and quality of the content are variable. Healthcare professionals should encourage patients to critically evaluate online resources. Implementing this strategy could enable patients to make more informed decisions about their treatment and, subsequently, better clinical outcomes.

**KEYWORDS:** Health literacy. Patient education as topic. Social media. Suburethral slings. Urinary incontinence.

José Maria Soares Júnior

*Scientifıc Editor*

